# Establishing proforma reporting in a busy DGH

**DOI:** 10.1186/1470-7330-15-S1-O2

**Published:** 2015-10-02

**Authors:** Sasha L Houghton

**Affiliations:** 1 Department of Radiology, Maidstone and Tunbridge Wells NHS Trust, Maidstone Hospital, Hermitage Lane, Maidstone, Kent ME16 9QQ, UK

## 

Maidstone and Tunbridge Wells NHS Trust is a large acute trust serving around 500,000 people in West Kent and North East Sussex. It is also home to the Kent Oncology Centre, which provides oncology care to around 1.8 million people across the whole of Kent and a large part of Sussex.

As a radiology department we had already established the use of proforma reporting for rectal cancers several years ago. We became involved in the RCR CaSPaR project [1] in 2012 and from that established the use of proforma reporting for both cervical and endometrial cancers, which we have continued to the present time, beyond the closure of the pilot. We have also independently developed a proforma report for MRI brain studies performed from our memory clinic as a response to an audit from the memory clinic team, which showed our reports were lacking critical information needed to secure approval for drug funding.

With the help of our PACS team we have embedded the proforma reports into the “text box” function of our GE RIS as word documents. The proforma is then filled in and edited as appropriate by the reporting radiologist.

The use of proforma reporting represents a significant change in reporting style, adopted by some more easily than others, as with any shift in practice. Managing this is a challenge, along with the IT issues. Furthermore, cancers where the diagnosis is known before the staging imaging is undertaken (e.g. colorectal and gynaecological malignancies) lend themselves more easily to proforma reporting than others where the diagnosis is often made after the imaging is performed, as in lung cancer. However the benefits of proforma reporting, which undoubtedly improves data collection, cannot be ignored and we should therefore continue to explore how best we can implement this change.
